# Continental Notes from a Holiday Correspondent

**Published:** 1894-08-18

**Authors:** 


### Continental Notes from a Holiday Correspondent.

### WIESBADEN.

Wiesbaden has become in recent years the most popular of the German watering-places, being visited by over 100,000 persons annually. It is easily reached, being about half an hour by rail from Frankfort-on-the-Maine, and two or three miles from the Rhine. The situation is decidedly good; it is on the southern spurs of the Taunus mountains, about 400 feet above the sea level, and protected from the north and east by these mountains, which are about 2,000 feet high. The climate is so good that Wiesbaden has attained considerable repute as a winter as well as a summer health resort. The town itself has about 70,000 inhabitants, is clean and well drained, and has an abundant and pure water supply. The streets are broad and well planted with shade trees; and hotels and lodging-houses are numerous, comfortable, and moderate in charges.

Its reputation depends on its hot springs, which have been known since Roman times. The chief of these is the Kochbrunnen, from which there is a flow of one hundred gallons of water per minute at a temperature of 144 deg. Fahr. Besides this spring there are twenty-three other springs, most of them belonging to hotels, and the total flow from these is about two hundred gallons per minute, giving the enormous total of three hundred gallons per minute for all the springs. The principal constituent of the water is sodium chloride, which is present to the extent of about seven parts in a thousand by weight. Potassium, calcium, and lithium salts are also present in appreciable quantities. The total quantity of salts discharged by these springs is about four and a half tons per day. The water is drunk to a comparatively small extent; it is chiefly used for bathing. There are eight bathing establishments supplied from the Kochbrunnen, and various smaller ones in connection with the hotels supplied by other springs; there are in all about nine hundred bath-rooms in Wiesbaden supplied with the hot mineral water. Most of these have jet, douche, and spray arrangements, as well as the ordinary plunge bath.

The chief beneficial use of these waters is in chronic rheumatism and gout. In his treatise on "The Nature and Treatment of Gout," Professor Ebstein, of Gottingen, says, "The treatment of gout by a course of saline water drinking has been too long neglected, especially that of the milder saline springs, among which those of Wiesbaden take a foremost rank, being also well adapted for a combined treatment with bathing, while the prevailing climatic and sanitary conditions favourably assist the cure. It has been demonstrated conclusively that the secretion of urine, and the elimination of uric acid, is promoted in a far greater degree by the waters of Wiesbaden than by those of the Carlsbad Mühlbrunnen." Dr. Kronecher, Professor of Physiology in Berlin, speaks of the Wiesbaden water as a "physiological tissue-rinsing fluid," as it contains nearly the same proportion of saline matter as the serum of the blood does.

The centre of social life in Wiesbaden, as in other German watering-places, is at the Kurhaus, where there are concert and reading-rooms, restaurant, &c. Visitors obtain admission to this, and all the concerts, &c., in it, to the Trinkhalle, where the waters may be drunk, and to all the public gardens, &c., by obtaining a visitor's ticket, which is given free to medical men, while to others it costs 1s. for the day, 15s. for six weeks, or 30s. for a year. Family tickets are also issued at reduced rates. The baths are paid for separately, and are generally taken in the hotel where one may be staying.

Though gout and rheumatism are the chief indications for a course of bathing at Wiesbaden, sufferers from many other ailments go there, and specially from catarrhal affections of the mucous membranes and digestive organs. In autumn the grape cure is in great vogue; it is prescribed for dyspepsia and obstinate chronic constipation. Several pounds of grapes are eaten daily, so that there is a great increase in the secretions. The appetite is said to be increased by it.

There are about a hundred medical men in Wiesbaden, among whom are specialists of every description. The one best known in England is Professor Pagenstecher, who I had the pleasure of seeing. He has a large private hospital in the Taunus Strasse, close to his own house; and a public hospital, of which he is the head, is close by. In the latter about 2,600 patients were seen last year, and 547 operations were performed. These included 147 cataract operations of all sorts. The majority of uncomplicated cateracts Professor Pagenstecher does without iridectomy; he did fifty last year, with forty-nine good, no medium, and one bad result. It is a pity that in all such reports the terms "good," "medium," and "bad," are not clearly defined, for until they are, it is quite impossible to judge the comparative merits of different operations.

It is not so much for his operations, however, that Professor Pagenstecher has become known, as for his success in the treatment of deep seated inflammation of the eye. His favourite treatment seems to be vigorous inunction with mercurial ointments and sweating treatment with hot baths. He has quite given up pilocarpine. He has the great advantage of having such cases in his hospital, where treatment is thoroughly carried out. He has many patients from the wealthier classes in England, and, like Father O'Flynn, he "has such a way with him" that he contrives to get his strictest orders obeyed. Whether, apart from the advantage of having his cases in his hospital, and constantly under his eye, his results are better than those of English surgeons is open to doubt.

